# Macular Rash as a Presenting Symptom of Acute Q Fever: A Case Report

**DOI:** 10.1155/crdi/9263690

**Published:** 2025-10-23

**Authors:** Muhammad Ali Muslimani, Valeria Brazzelli, Marco Lucioni, Angela Maria Di Matteo, Raffaele Bruno, Enrico Brunetti

**Affiliations:** ^1^Department of Clinical, Surgical, Diagnostic and Pediatric Sciences, University of Pavia, Pavia, Italy; ^2^Infectious and Tropical Diseases, Fondazione IRCCS Policlinico San Matteo, Pavia, Italy; ^3^Institute of Dermatology, Fondazione IRCCS Policlinico San Matteo, Pavia, Italy; ^4^Department of Molecular Pathology, University of Pavia, Pavia, Italy; ^5^Anatomic Pathology Unit, Fondazione IRCCS Policlinico San Matteo, Pavia, Italy

**Keywords:** *Coxiella burnetii*, coxiellosis, cutaneous manifestations, eruption, erythema, exanthem, infectious, parainfectious, Q fever, rash

## Abstract

**Background:**

Q fever is a globally distributed zoonotic infection caused by *Coxiella burnetii*, exhibiting a broad clinical spectrum in both acute and chronic forms. While pneumonia, hepatitis, and endocarditis are well-recognized manifestations, cutaneous involvement remains poorly characterized and likely underreported.

**Case Presentation:**

A 63-year-old male metalworker was admitted with a 2-week history of high-grade fever, dyspnea, anorexia, intractable hiccups, and profound asthenia. Physical examination revealed a diffuse, nonpruritic, blanchable macular rash on the back and sacral region. Extensive microbiological and autoimmune investigations were negative. Chest imaging demonstrated bilateral pneumonia, mediastinal lymphadenopathy, and a small pericardial effusion. Skin biopsy showed mild acanthosis, dermal capillary congestion, and superficial lymphohistiocytic infiltrates. Serological testing confirmed acute Q fever, with elevated Phase II *C. burnetii* antibody titers. Oral doxycycline led to complete resolution of fever and rash. Serial serology demonstrated a progressive decline in antibody titers, and the patient remained symptom-free after 6 months.

**Discussion:**

This case highlights a rare parainfectious macular rash associated with acute Q fever—apparently the first reported in Italy. Cutaneous involvement in Q fever may represent a parainfectious immune-mediated reaction. Its nonspecific appearance and lack of a characteristic distribution pattern often delay diagnosis.

**Conclusion:**

Clinicians should maintain a high index of suspicion for *C. burnetii* infection in patients with unexplained fever, pneumonia, and rash, even in the absence of direct animal exposure. Multidisciplinary evaluation and serial serology are pivotal for timely diagnosis, effective management, and monitoring of disease resolution.

**Summary**

• Q fever is a worldwide zoonosis which exhibits a diverse clinical spectrum in both its acute and chronic forms.
• Cutaneous manifestations seem to be underreported.
• No distinguishable pattern of body distribution has been so far described.
• Clinical suspicion and a thorough evaluation considering the patient's history, symptoms, and potential exposure to *Coxiella burnetii* are crucial for accurate and timely diagnosis.
• Serial serological testing is useful in monitoring the resolution of an ongoing *C. burnetii* infection.
• As Q fever's reemergence poses challenges in diagnosis and management, a multidisciplinary approach can help shorten the time to therapy and improve clinical outcomes.

## 1. Introduction

Q fever, or coxiellosis, is a worldwide zoonosis caused by the intracellular bacterium *Coxiella burnetii* (*C. burnetii*) which can present both in acute and chronic forms. The clinical manifestations of acute Q fever include endocarditis, pneumonitis, and hepatitis, whereas skin involvement is rarely described. We report a case of acute Q fever presenting with atypical pneumonia and a peculiar parainfectious macular rash. To the best of our knowledge, this is the first reported case of a parainfectious Q fever–related rash in Italy.

## 2. Case Presentation

**Figure 1:**
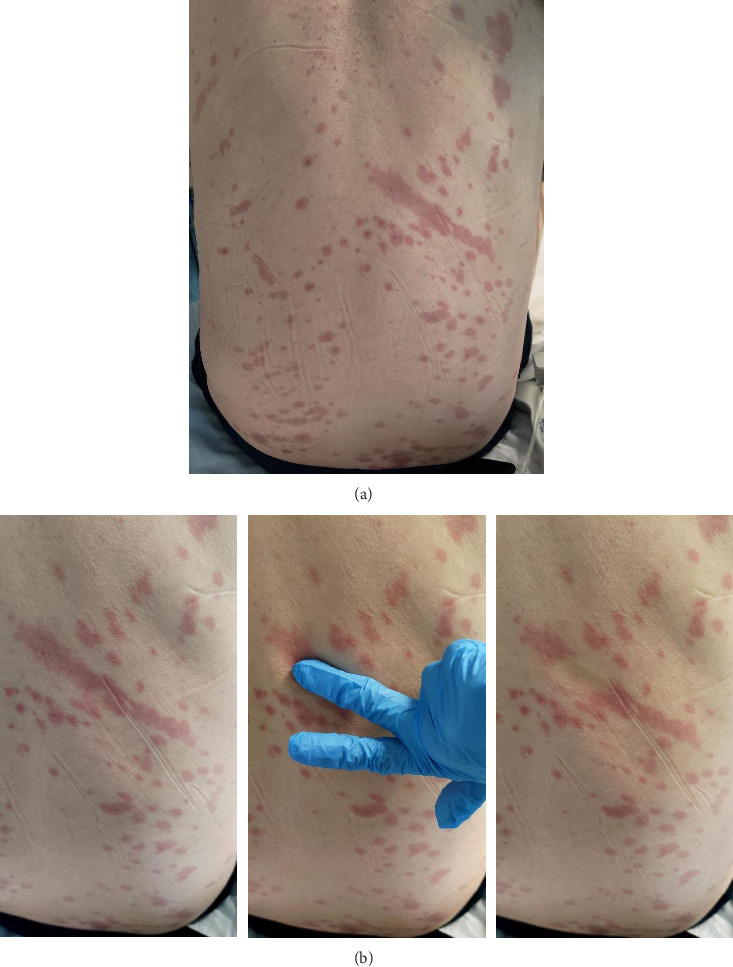
(a) Initial presentation with asymptomatic diffuse red macular eruption all over the back. (b) Blanchable macules on the back.

A 63-year-old male metalworker was admitted to our hospital because of a 2-week high-grade fever and dyspnea, associated with anorexia, incoercible hiccups, and profound asthenia. He denied direct contact with animals or consumption of unpasteurized dairy products. On inspection, the patient showed diffuse painless nonpruritic red macular eruption all over the back down to the sacral region (Figure 1(a)). The macules were blanchable (Figure 1(b)) and occasionally coalescent. There was no desquamation or induration. Nikolsky's sign was negative. Except for appendectomy and surgical ileal resection with ileo-ascending LL anastomosis for volvulus, his medical history was unremarkable for chronic conditions, medication, or substance abuse. He had no known allergies and had never experienced adverse drug reactions.

Given the nonspecific clinical presentation, a broad differential diagnosis was considered. The antigenic test for SARS-CoV-2 returned negative. Three distinct sets of blood cultures were negative. Urine (*Legionella pneumophila* antigen, *Streptococcus pneumoniae* antigen, West Nile virus RNA, pan-Flavivirus RNA, and bacterial/fungal culture) and stool (*Clostridioides difficile* GDH antigen, bacterial culture for *Shigella*, *Campylobacter*, and *Salmonella* spp) test panels were negative. Multiplex PCR testing for all seasonal respiratory viruses on a nasopharyngeal swab was negative. Comprehensive tests for autoimmune diseases were also negative. Furthermore, serology and molecular tests were negative for human immunodeficiency viruses 1 and 2, monkeypox virus, human parvovirus B19, tick-borne encephalitis (TBE) virus, West Nile virus, and the eight human herpesviruses. An incisional skin biopsy was performed.

**Figure 2:**
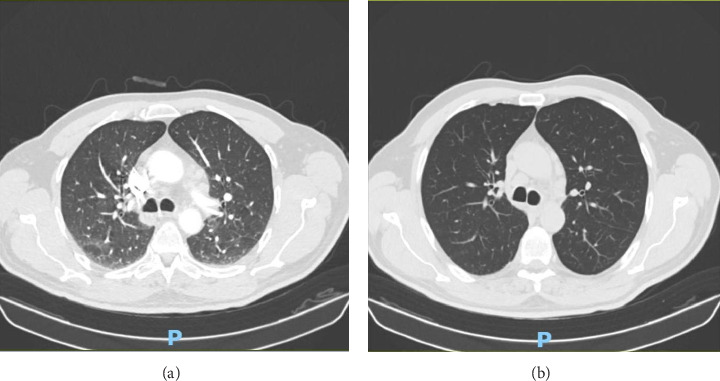
Thoracic CT scans: at baseline (a) revealing bilateral pneumonia and at 14 days (b) revealing radiological resolution.

A lung x-ray documented bibasilar dysventilation, blunting of the left costophrenic angle, and minimal congestion of the small circulation. Contrast-enhanced Computed Tomography (CT) scanning showed bilateral pneumonia and revealed diffuse mediastinal lymphadenopathies as well as a 12-mm pericardial effusion (Figure 2). Fluorodeoxyglucose-Positron Emission Tomography (FDG-PET) further highlighted the presence of reactive cervical and mediastinal lymphadenopathies but did not detect tumors or neoplasms. Optical bronchial fibroscopy and microscopic/culture examination of bronchoalveolar lavage revealed no microorganisms, especially alcohol-resistant bacilli.

Hematology, pulmonology, and thoracic surgery consults excluded conditions of relevance. Both the pneumonia and the rash did not respond to systemic empiric antibiotic therapy with piperacillin/tazobactam 4.5 g QID and levofloxacin.

**Figure 3:**
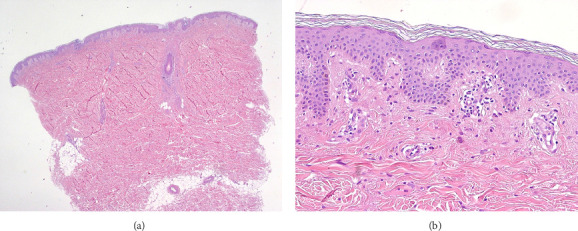
Incisional skin biopsy showing mild acanthosis and hyperkeratosis ((a), HE 2X) and minimal superficial perivascular infiltrates consisting of small lymphocytes and histiocytes ((b), HE 20x).

Histological examination (Figure 3) of the incisional skin biopsy showed mild acanthosis and hyperkeratosis, with congestion of dermal capillaries and minimal perivascular, superficial lymphohistiocytic infiltrates.

Given the lack of response to empiric antibiotic therapy that did not cover intracellular pathogens, an infection by such bacteria was suspected. A dedicated serology panel was thus carried out.

Quantitative serology testing using chemiluminescent immunoassay (CLIA) (VirClia, Vircell) revealed high Phase II antibody titers to *C. burnetii* antigens (IgM 2.37; IgG 1.87 [< 0.9 negative; 0.9–1.1 dubious; > 1.1 positive]). Liver needle biopsy was then performed, and histological examination excluded hepatic involvement. Similarly, liver biopsy cultures were negative. To exclude infective endocarditis, a transthoracic echocardiogram was carried out, which ruled out valvular vegetations.

**Figure 4:**
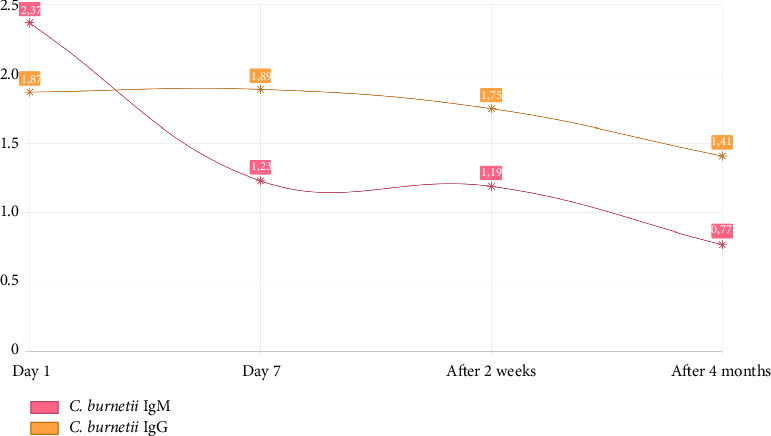
Serial quantitative serology against *C. burnetii* antigens (< 0.9 negative; 0.9–1.1 dubious; > 1.1 positive).

The patient was then treated for acute Q fever with oral doxycycline 100 mg BID for 14 days. He recovered gradually, with resolution of the fever as well as clearance of the rash. Serum antibody titers to *C. burnetii* antigens were serially dosed (Figure 4), showing progressive decrease in titers. After 6 months of clinical and laboratory follow-up, the patient remained disease-free and required no further therapy.

## 3. Discussion

*C. burnetii* infection was first identified by Edward Holbrook Derrick in 1935, and the pathogen was later named after Dr. Herbert Cox and Dr. Burnet. Because of its exceptionally infectious nature and an airborne route of transmission, this bacterium is a potential agent of bioterrorism. The Centers for Disease Control and Prevention (CDC) classifies Q fever as a Category B agent. It preferentially infects livestock animals, which act as a reservoir for the pathogen. Humans usually acquire the infection through occupational exposure or contact with infected animals or their products. Transmission to humans occurs through inhalation of contaminated aerosols, ingestion of contaminated food products, or direct contact with infected animals. The incubation period ranges from 2 to 3 weeks. The clinical manifestations of Q fever may be so variable that the disease is often diagnosed only if it has been systematically considered. Prompt recognition and treatment are imperative to prevent complications and minimize morbidity associated with the disease. Treatment chiefly involves antibiotics, typically tetracyclines to which no significant resistance has been so far observed, in combination with other medications depending on the severity of the disease. Effective vaccines exist for humans, are sometimes offered to at-risk populations, but are currently still unavailable for the wider population in most countries [1–4].

While the patient's clinical presentation was primarily compatible with an infectious process, autoimmune or malignant conditions were not promptly excluded. Whereas the symptoms were readily explainable, the hiccups were attributed to mediastinal lymphadenomegaly that most likely irritated the phrenic nerve. This symptom resolved as soon as the lymph nodes regressed in size. Laboratory investigations showed high inflammation indices (C-reactive protein 15.56 mg/dL [< 0.5] and procalcitonin 12.1 ng/mL [0.00–0.50]), while imaging documented cervical and thoracic localization of reactive inflammatory response marked by diffuse characteristic lymphadenopathy. Blood cells subpopulations were analyzed, and no single pathological expansion was detected. After specialist assessment, both autoimmunity and malignancy were excluded.

The serological positivity for *C. burnetii*–specific antibodies and the subsequent improvement with targeted antibiotic therapy confirmed the diagnosis of acute Q fever, which in this case predominantly involved the lungs. From an epidemiologic perspective, the patient worked as a smith and a metalworker, which meant he was at no direct occupational risk. However, further investigations indicated that the patient lived in a geographical area entirely surrounded by cattle farms and dairy production facilities. It is well established that infectious aerosols can travel long distances affecting people living downwind of an infected cattle farm [5]. In accordance with the regional infectious disease monitoring program instructions, the case was reported to the local health surveillance authorities. In 2023, reported cases of human Q fever in the EU occurred year-round, with 805 confirmed cases, an increase of 11.5% compared with 2022. However, the overall trend did not show any significant increase or decrease in the 2019–2023 period [6].

Q fever, in both its acute and chronic forms, exhibits a diverse clinical spectrum. While the most common are flu-like symptoms, pneumonia, hepatitis, endocarditis, and rarely, osteomyelitis [7–11], skin manifestations have also been reported. It is important to note that the infection can be asymptomatic [9].

In the literature, skin manifestations have traditionally not been considered a primary feature of Q fever [12]. Nevertheless, in certain subsets of patients, such as in children, the prevalence of exanthem-like rash is as high as 50% of diagnosed cases [13,14]. In fact, in addition to fever and gastrointestinal symptoms, skin signs are specifically predominant during the acute phase of Q fever in children [9]. Interestingly, it has been suggested that cutaneous manifestations might be more frequent in chronic Q fever cases, regardless of the age group [15]. Skin involvement can occasionally occur as an isolated symptom of Q fever [9].

The most frequently reported cutaneous manifestations (Table 1) comprise erythema multiforme, erythema nodosum, maculopapular eruptions, purpuric lesions, and small-vessel vasculitis. In up to 20% of cases, Q fever is associated with nonspecific exanthems, most commonly a maculopapular rash on the trunk [20]. Maculopapular rashes, characterized by flat and raised red lesions, often resemble measles or scarlet fever rashes. Erythema nodosum, marked by tender red nodules, appear predominantly on the lower extremities. Yet there seems to be no unique lesion localization, as most body regions have been reported to be impacted, and no distinguishable pattern of compartment distribution has been so far described.

The pathophysiology underlying cutaneous manifestations in Q fever is still largely unknown, but it is suspected to be immune-mediated, involving an intricate interplay between the host's immune response and the bacterial pathogen. It is established that *C. burnetii* possesses unique cell-wall components and effector molecules that trigger host immune responses, leading to cytokine release and activation of immune pathways [23]. This dysregulated immune response could plausibly induce various cutaneous manifestations.

The predominance of Phase II antibodies in the initial serological assessment confirmed the acute nature of the disease. Phase II IgM antibodies are detected first, usually around Day 7 from the onset of symptoms, followed by the appearance of IgG antibodies about 5 days later. Ideally, IgA-class antibodies should be assayed during the initial phase of the infection [24]. At the time, this was not feasible in the hospital's laboratory and was then deemed inessential in terms of establishing the diagnosis. For the same reason, Polymerase Chain Reaction (PCR)–based assays of biological material or the biopsied tissues were not further sought.

In conclusion, cutaneous involvement appears to be most likely overlooked and underestimated in Q fever. Diagnosing cutaneous Q fever manifestations can be challenging due to their lack of specificity and resemblance to other infectious and noninfectious skin conditions. Clinical suspicion and a thorough evaluation considering the patient's history, symptoms, and potential exposure to *C. burnetii* are crucial for accurate and timely diagnosis. Serological tests, specific PCR, and cell culture can provide evidence of an ongoing *C. burnetii* infection, but direct cutaneous findings should raise clinical suspicion and warrant histopathological examination. Consulting a dermatologist can help shorten the time to diagnosis. As Q fever's reemergence poses challenges in diagnosis and management, further research into its cutaneous manifestations can enhance our understanding of the disease and improve clinical approaches.

## Figures and Tables

**Table 1:** Summary of the cutaneous manifestations of Q fever reported in the last 4 decades.

Authors	PMID	Year	Country	Q-fever form	Patient gender	Patient age (years)	Cutaneous manifestation(s)
Jutraž et al. [16]	37365895	2023	Slovenia	Acute	Male	42	Erythema exudativum multiforme (EEM)–like exanthem
Lencastre Monteiro et al. [17]	34853746	2021	Portugal	Acute	Male	59	Maculopapular, nonpruritic, symmetrical exanthem
Meriglier et al. [18]	28819912	2018	France	Acute	Female	37	Erythema nodosum
Koh et al. [19]	29028126	2018	United States (California)	Acute	Male	41	Leukocytoclastic vasculitis
Boele van Hensbroek et al. [20]	10783031	2000	Netherlands	Acute	Male	4	Maculopapular rash and generalized petechiae
Betlloch et al. [21]	1769774	1991	Spain	Acute	Male	24	Erythema annular centrifugum
Enzenauer et al. [22]	2023203	1991	United States (Colorado)	Chronic	Female	41	Purpura

## Data Availability

The data that support the findings of this study are available from the corresponding author upon reasonable request.
